# ‘*They want to get rid of us by all means*’: A critical analysis of policy and governance responses and their implications for street traders’ access to urban space in Harare, Zimbabwe

**DOI:** 10.1177/23996544241298414

**Published:** 2024-11-06

**Authors:** Elmond Bandauko, Godwin Arku

**Affiliations:** 6221 The University of Western Ontario, Canada

**Keywords:** Urban informality, urban governance, urban space, street trading, Harare

## Abstract

This paper examines how urban authorities in Harare respond to street trading and the implications of these interventions on street traders’ access to urban space. Drawing from focus groups, in-depth interviews with street traders and urban governance actors, we argue that as urban authorities in Harare become obsessed with defending modernity, they implement aggressive urban policies aimed at eradicating street traders’ livelihoods. These violent measures are often differentially experienced with ‘street vending mothers’, the elderly and those with disabilities bearing the brunt of Harare’s authoritarian spatial governance. Alternatively, the city has also experimented with what we call governing through ‘spatial containment’, aimed at ‘taming’ street traders and transforming them into formalized entities. We demonstrate that despite its noble intentions, such a policy approach has unintended outcomes for street traders since it undermines the organic attributes of their trade: operational flexibility, spatial mobility and proximity to customers. Despite the negative implications of policy interventions, street traders’ associations struggle to champion the collective voice of informal traders due to their organisational fragmentation, unfavourable political environment and existing structural constraints. This study contributes to the broader scholarly debate on urban informality, governance, and socio-economic justice in developing country contexts. Based on our findings, we call for a paradigm shift in policy and governance approaches, advocating for inclusive urban planning, dialogue, and recognition of the socio-economic contributions of street traders.

## Introduction

On that bright Monday morning in May 2022, the first author entered the Office of the Senior City Planner at Cleveland House, nestled in the heart of Harare’s Central Business District (CBD). As the first author’s eyes scanned the room, he quickly noticed a captivating land use map, displayed on the Planner’s desk. Fueled by curiosity, the first author could not resist the urge to inquire about the map’s significance. The Principal Town Planner revealed the map’s purpose:

You see young man; this is a land use map of local development plan 22 which covers the Harare CBD. We are in the process of reviewing and updating the local plan and rationalising land uses because a lot of things have changed since this plan was approved in the 1990s…The function of the Town Planning Department is to make sure land uses are equitably distributed and to make sure that every citizen benefits from the space in the city and that it all happens in an orderly manner.

Interestingly, the sentiments by the Principal Town Planner directly aligns with the objectives of our research. Our interest in interviewing officials from City of Harare’s Planning Department was to solicit their perspectives on the planning and governance of urban space; one of the complex urban challenges confronting the city today. When we asked the Senior Planner to describe how the city manages street trading, she highlighted that:

Though the City has been tolerant to street trading, there are times when we resort to evictions. We have no choice but to clean up urban spaces so that the city is not overtaken by street vending. Sometimes, the vendors turn the city upside down because they create several problems such as blocking traffic and pedestrian flows. Street vending by its character is an invasion of urban space…. (Principal Town Planner).

From the above narrative, the City of Harare currently faces a difficult task of efficiently and effectively planning and governing public space due to competing demands from multiple users. When we asked the Principal Town Planner to explain what they meant by street traders ‘turn the city upside down’, it was highlighted that street traders have significantly increased in the city center, creating intense competition for space between different users and thus generating complex challenges for city managers. Her sentiments demonstrated that as a planner, she perceived street traders as a ‘problem’ that must be fixed. According to the Town Planner, the challenge for city officials is to balance these competing interests, so that the conflicts between the multiple users are resolved. However, the planner acknowledged that this is not an easy thing to do. Our encounter with the Town Planner resonates with most Global South cities since street trading has become a distinctive feature providing livelihoods for billions of the urban poor who struggle to be accommodated in formal labour markets. Despite being a visible and important aspect of urban life, it remains a complex phenomenon for city officials, who are struggling to balance the need to manage public space and support livelihoods of the urban poor.

Consequently, the governance of street trading has become a contentious issue and it has attracted scholarly attention, public debate, and policy interventions (Bénit-Gbaffou, 2016; Huang et al., 2019; Kamete, 2013a; Kamete, 2013b). Most of the urban studies literature in Global South cities highlights how colonial legislation and planning instruments have continued to shape urban development in post-colonial times (Kamete, 2013a; Watson, 2009). These instruments often prioritize aesthetics, efficiency, and modernization, while neglecting the needs and interests of the urban poor. One consequence of this approach is that street trading is frequently viewed as a hindrance to the modernization of cities and subject to aggressive regulation. Some scholars argue that that urban redevelopment policies aimed at ‘recovering’ and reimagining public space have further marginalized and excluded the poor (Adama, 2020; Crossa, 2009). These policies often involve the eviction of street vendors, and the introduction of new forms of surveillance and control in public spaces. These measures are justified on the grounds of protecting the image of cities and create favourable climate for investment. Urban scholarship has made significant strides in exploring how exclusionary urban practices impact the livelihoods of street traders. Most of the urban scholarship largely focuses on Asian and Latin American cities, where neo-liberal and entrepreneurial urban governance models have been implemented with diverse consequences on the urban poor (c.f. Crossa, 2009, 2016).

However, despite these advancements, there are still research gaps that need to be addressed. Majority of existing studies do not adequately investigate the differential impacts of these exclusionary governance practices on different groups of street traders. Such studies fail to recognize that street traders are not a monolithic group, but are instead diverse and their experiences in contested urban spaces are influenced by various characteristics, such as gender, age, and trading experience, among other factors (Adama, 2020; Crossa, 2016). Additionally, what is currently missing and urgently needed is “a critical, qualitative focus on the experiences of women street vendors, the gendering of vending spaces, and a socio-spatial analysis grounded in the Southern urban context” (Saxena, 2024, 2024: 2). Therefore, the “study of gender-differentiated experiences of vendors is essential to gain deeper insights into urban informal street spaces” (Wang et al., 2024: 50). Beyond gender, ‘accounting for other social conditions’ such as age and disability is also critical since street traders are a heterogenous social group whose concerns, backgrounds and experiences are highly differentiated. Street traders by their very nature are “internally differentiated, resulting in different degrees of exclusion, power, resources, mechanisms of exclusion, and practices of negotiation and resistance” (Crossa, 2020: 170). Policy and governance responses to street trading are implemented in variegated national and urban political economies, which make each geographic context unique, and therefore requiring contextualized studies. Studies on urban governance and street trading have predominantly centered on analyzing the negative consequences of overly confrontational practices such as street traders evictions, often overlooking the complexities of alternative policies such as efforts towards the spatial formalization of street trading (Huang et al., 2019). While exclusionary practices, such as evictions and punitive regulations, indeed highlight the adversities faced by street vendors, it is equally important to consider the potential benefits and challenges associated with efforts aimed at spatial formalization.

To address these research gaps, this paper engages with the following question: how do urban authorities in Harare respond to street trading and what are the implications of these interventions on street traders’ access to urban space? Ultimately, this paper aims to contribute to a more nuanced understanding of how urban policy and governance shape the experiences of street traders who rely on access to public space for their livelihoods. The findings reveal the need for a holistic approach that considers the social, economic, and spatial dimensions of street trading to promote sustainable and equitable development as promoted in the New Urban Agenda (NUA) and Sustainable Development Goal #11 on inclusive cities.

The rest of the paper proceeds as follows: In the next section, we provide an overview of the theoretical debates on governing street trading. This is followed by an outline of the study context and the methodological approach adopted for the study. Next, we present the results based on our empirical research in Harare. In the last section of the paper, we discuss the research findings and highlight their implications for urban policy and governance.

## Governing street trading: Theoretical perspectives

Governing street trading is a complex and multilayered process, often involving multiple actors with conflicting rationalities and competing interests (Lindell, 2019). Indeed, livelihood spaces in rapidly urbanizing cities of the Global South are socially produced by heterogenous and contingent configurations of differently powerful urban actors. Theoretical debates on governing street trading have been heavily contested in urban studies literature (Bénit-Gbaffou, 2016; Huang et al., 2019; Lindell, 2019). Among scholars of urban geography, Foucault’s theory of governmentality has become prominent in exploring multiple power-seeking practices and interventions that are aimed at guiding actions, behaviours and inner states of others (Huang et al., 2019). Governmentality refers to the way in which the state exercises control over the population and the various techniques and strategies it uses to govern. The term combines ‘government’ and ‘mentality’, indicating a focus on the mentalities and rationalities that underpin the practice of governing (Garmany, 2010; Huang et al., 2019; Huxley, 2008).

Theoretically, governmentality has been deployed by urban researchers to understand governments’ responses to street trading. For instance, spatial regulations designed to keep street traders out of certain public spaces are more centrally linked to prevailing urban governance paradigms in Global South cities (Roever, 2020). The ways in which these paradigms appear in policy and practice vary, ranging from permit regulations that are specific to certain areas and determine who is allowed to sell in which location, to relocation initiatives aimed at transitioning street vendors to off street commercial spaces, to even large-scale evictions. Lindell (2019) reiterates that most Global South cities are undergoing a shift where various urban authorities use different approaches of governance based on the current political and economic conditions. These governing modes include strategies such as the establishment of vending free zones or by simply moving the ‘problem’ of street trading to other spaces in what scholars describe as ‘spatial enclosure’ (Lindell et al., 2019). For instance, in Bogota, Colombia, a transition towards municipal socialism resulted in perceiving street vendors as workers rather than entrepreneurs. This change in perspective, supported by constitutional court decisions in favour of the vendors’ right to work, facilitated the implementation of more progressive policies aimed at promoting their livelihoods (Linares, 2018). Other cities like Guangzhou, China have implemented formalization as a spatial strategy by putting street traders into designated spaces. The relocation of street traders to designated markets has often been associated with better management of vendors and urban spaces by urban institutions. Some scholars describe the process of creating alternative spaces as “governing spatially”, where urban authorities use power to reconfigure space (Huang et al., 2019; Lindell et al., 2019; Morris, 2022). Huang et al. (2019) argue that formalization creates bounded spaces that are subject to state regulation and control, such as designated marketplaces or street vendor zones. These spaces are designed to guide the behaviour of informal economic individuals towards officially desired norms and standards, such as hygiene, safety, and tax compliance.

Furthermore, one of the most extreme forms of governance responses to street trading is large scale eviction, where street traders are violently removed from public space (Roever, 2020). The violent removal of street traders from public spaces is rationalized by the need to create ‘modern cities’ that are characterized by order and aesthetics (Bandauko and Arku, 2024a). These punitive urban policy interventions have been characterised as a form of urban revanchism (Huang et al., 2014; Swanson, 2007), which denotes vengeful reaction against the urban poor as dominant authorities attempt to ‘tame the wild city’ and bring it under the control (Smith, 1996). To ‘tame the wild city’, urban officials often use a combination of the following revanchist measures: punitive ‘purification of public spaces’, criminalization of street economies, zero tolerance of ‘undesirables’ and state-led production of space, where new forms of structural violence are activated. Originally a phenomenon of the Global North, urban revanchism is now increasingly visible in cities of the Global South, where governments are implementing urban “cleansing” programs to rid their cities of perceived nuisances and out-of-place elements. In many Global South cities, urban revanchism often targets vulnerable populations like informal settlers and street vendors, viewing them as obstacles to urban modernity (Makanadar and Bandauko, 2024). These theoretical insights provide a valuable framework for examining the policy and governance responses to street trading in Harare, Zimbabwe. Like many other cities in the Global South, Harare has employed diverse governing technologies, each with its own rationale and outcomes, leading to both positive and negative impacts on the livelihoods of street traders.

## Study context

This study was conducted in Harare, Zimbabwe’s political and administrative capital. Harare has an estimated population of over 2 million residents. Specifically, we conducted our research in Harare’s Central Business District (CBD). As an epicenter of economic activity, the CBD attracts many street traders due to high customer base (e.g., office workers, shoppers, tourists, and residents), market accessibility and access to public spaces. At the same time, the CBD is the focal point for municipal surveillance and control as it symbolizes the projected image of Harare and the nation at large. While these public spaces are strategic for street traders with respect to access to high volume of customer flows, they also generate frictional encounters between street traders and urban authorities who are preoccupied with promoting their vision of a ‘modern’ city. However, there is a huge discrepancy between Harare’s vision and realities on the ground (Bandauko and Arku, 2024b; Karakadzai et al., 2023). For the better part of the twentieth century, compared to other cities of the Global South, Harare had few permanent large-scale illegal land use activities. However, the situation has drastically changed since the 1990s due to the implementation of Economic Structural Adjustment Programme (ESAP), which resulted in mass retrenchments and rising cost of urban living (Kamete, 2012). From 2000 onwards, many individuals turn to the informal sector as a means of survival.

As the Zimbabwean economy constantly deteriorates, different groups of street traders including women, young people, the disabled and elderly appropriate public spaces, selling different products such as vegetables, fruits, electronics, clothes, cosmetics, and food items among others. Statistics on street traders are rarely captured, but conservative estimates suggest that there are currently more than 20000 street traders operating in Harare’s CBD (Matamanda et al., 2020). This figure is grossly underestimated given that the population of street traders in Harare has recently skyrocketed due to dwindling employment prospects and rising urban poverty for most of urban residents. Street trading in Harare is largely dominated by women (Bhila and Chiwenga, 2023). However, the street traders operate in a very hostile and repressive context where they are the primary target of displacements in the name of ‘purifying’ public spaces and maintaining urban order (Bandauko et al., 2021). Additionally, Street traders are often stigmatized as a ‘nuisance,’ criminalized, discriminated against, and subjected to multiple social and economic insecurities (Bandauko and Arku, 2024a). Therefore, our research context is characterized by rising urban poverty and marginalization, where street traders’ livelihood practices are disenfranchised (Bandauko and Arku, 2023).

The City of Harare has a plethora of policy and governance instruments that are mobilized to govern street trading. The principal legislation, the Regional, Town and Country Planning Act, RTCPA (Chapter 29:12) provides the framework for the planning of local areas with the objective of *conserving and improving the physical environment and in particular promoting health, order, safety, and amenity*. The Act also grants powers to urban councils to regulate the appearance of urban landscapes as well as control over development including the use of land and buildings. The CBD falls under City Center local development plan 22. The local development plan defines land use categories and zoning regulations for different areas within the city center. It identifies areas for commercial activities, residential development, mixed-use zones, public spaces, and other designated land uses.

## Data and methods

This paper is part of a larger project that investigates how contemporary modes of urban governance in Harare are experienced, negotiated, and resisted by street traders in their pursuit of accessing and utilizing urban spaces. Data for this study was collected through a combination of qualitative methods such as key informant interviews (KIIs) including urban governance actors, semi-structured interviews and focus group discussions (FGDs) with street traders. We conducted a total of 26 KIIs with different groups of stakeholders including Planners from the City of Harare, Municipal Police Officers, Civil Society Leaders, Academics/Researchers on urban issues in Zimbabwe, Planners from the Ministry of Local Government among others. These key informants were purposefully selected based on their experiences and knowledge on urban informality, urban governance, and livelihoods of the urban poor in Harare. We held discussions with officials that are involved in the planning and governance of urban space (e.g., city planners), those that are engaged in everyday enforcement of municipal regulations such as by-laws (e.g., development control officers, municipal police officers) as well as local elected officials that are responsible for the ratification of council decisions regarding the use of urban space in Harare. The diversity of urban governance actors that we interviewed enabled us to explore different dynamics with respect to contestations, entanglements and the everyday struggles that characterise street trading in Harare.

We used a key informant interview guide covering different thematic issues such as (i) policy and governance on street trading, (ii) the relationships between street traders and urban governance actors, and iii) how urban policies and governance practices affect different groups of street traders in Harare. In addition to the key informant interviews, we also conducted semi-structured interviews with 19 street traders selected using purposive sampling. The selection criteria were based on the type of goods sold, location of trade, gender, and age. The interviews focused on the traders’ perspectives on urban policies and practices. Additionally, we conducted focus group discussions with a total of 3 groups of street traders, each comprising of 8 members. These groups include female only, young street traders and experienced street traders (those that have been operating for 10+ years). We analysed the data using inductive thematic analysis. After transcribing interviews and focus groups, we imported the data into NVivo software. The analysis followed four steps: familiarizing with the dataset through active reading, generating initial codes in NVivo, searching for themes within the coded data, and reviewing themes to ensure authenticity. This method provided a comprehensive understanding of the data, and the results are presented next.

## Results

### Street trading, ‘utopianization’ of urban space and conflicting city images

In Harare, there is a disconnect between city aspirations, planning systems and pervasive street trading. These conflicting uses and image of the city have always sparked debate in the city. Urban streets in Harare are objects of the disciplinary powers of the state and as such become a contested form of public space in which the daily practices of city inhabitants and the abstract ideals and goals of city leaders clash. This dissonance between the city’s aspirations and the realities of street trading has fueled a heated debate among urban planners and policymakers. Urban practitioners perceive street trading as a chaotic and unsightly practice that needs to be regulated, controlled and ‘tamed’ to bring the city in line with modernistic aspirations.

We are trying to sell our city to the outside market to attract investors, but we cannot attract investors in a state of chaos, in a state of disorder, in an environment of lawlessness. Remember, we have a vision to be a ‘world class city’ and a world class city largely is defined by the structure and form of the city. So, there is nowhere we can attain this vision when we have everyone doing anything anywhere. (Chief Development Control Officer, City of Harare)

For me, Harare’s world class city vision is about having order in everything that we do, where everything will resemble what happens in First world cities. It is about having urban spaces that are not contaminated by undesirable elements. (Senior Municipal Police Officer)

The above quotes accurately capture the tensions between aesthetics of Harare and the aesthetics of the informal street worker’s body. City officials from the planning department unapologetically aspire towards orderly planned and aesthetically vibrant urban environments, which mirror those from the western world. Indeed, most planners reiterated that Harare needs to restore its ‘glow’, lustre and vitality as Sunshine city that is attractive to investors. However, these ideals do not necessarily align with the lived realities of most street traders who operate in the informal economy, as reiterated by the key informants in our study. The places designated to convey a good image of Harare are also precisely those that are the most economically viable and profitable spaces for street trading. The different meanings of public space for the urban authorities and street traders express an underlying tension between world-class-city-making and livelihood practices of the poor.

I think Harare is coming from a static imagery of good urbanism. In Harare, we have planners who want an orderly city and who are aiming for classical cities like London and other cities in the West without taking into considerations the national and local context. So, if urban authorities are not cognizant of and turn a blind eye to the economic fundamentals and peoples’ everyday reality, there is a danger of designing policies, strategies and practices that do not align with how people experience urban life. (Urban Governance Practitioner)

Harare aspires to be a world class city. A world class city is an important node of globalization. London and Johannesburg are important nodes of globalization. So, Harare sees itself as an important and growing node of global capital. That is where they want to take themselves to. And that vision currently contradicts with vision of ordinary ‘Harareans’ who see the city as a space where they can trade and make a living. (Civil Society Leader 1)

From the above quotations, in Harare there is contestation between the desired city as conceived by city planners and the existing city as lived and experienced by the urban poor. The urban authorities’ vision of the city has no room for the ambulant informal proletariat who appropriate space in contravention of urban planning regulations. Thus, the way urban spaces are produced, designated, and allocated indicates that only Western-influenced practices and ways of living that conform to strict guidelines are deemed acceptable and permitted. The urban authorities’ fetishization of ‘modern cities’ resulted the implementation of violent crackdowns, what we describe as governing through aggressive repression.

### Governing street trading through aggressive repression: ‘*They want to get rid of us by all means*’

Urban authorities in Harare are inclined towards a governing mode based on aggressive repression. Aggressive repression is a declaration of ‘*spatial war*’ against street traders by state and is anchored on ‘*zero tolerance*’ to any form of informality. Municipal and state enforcement officers use their dominating and coercive power through authoritarian spatial governance, where municipal by-laws and land use regulations are vigorously enforced to eradicate and annihilate perceived nuisance. Under this mode of governance, abusive policing and violent encounters between street traders and enforcement authorities have become an order of the day in Harare’s urban spaces. Municipal and state police engage in a never-ending game of “*cat and mouse*” with informal traders. One FGD participant described the city’s approach to street traders as ‘ruthless’, ‘merciless’, ‘inconsiderate’ and ‘notoriously aggressive’. Since the implementation of the famous Operation Murambatsvina (Operation drive out fifth) in 2005, the City of Harare has implemented multiple clean up campaigns and blitz targeting primarily street traders, pushing them off the streets and denying them the right to physically occupy and appropriate urban spaces. One of the street traders highlighted that their everyday experience with the police is like ‘opening wounds’ of the brutal consequences of Operation Murambatsvina, which literally wiped out any form of informality in the city. The Harare (Vendors) By-Laws of 2020 gives powers to the city to order the removal of vendors in the interests of public health, public safety, town, and country planning as well as to demolish any illegal vending sites within twenty-four hours of giving notice. As a result, Harare’s CBD has become an intense battleground between informal traders and enforcement authorities. In extreme cases, the city center is heavily militarized through the deployment of the dreaded riot police unit to deter vendors from (re)appropriating urban space.

The state police when they provide back up to municipal police, they can be very brutal and ruthless in their operations. Their motive is to ‘wipe’ out the vendors from the streets of Harare. The government used to deploy state police to come and raid this street, because they considered us as ‘notorious’ vendors who do whatever they want without any consequences. (Simeon)

The ‘militarization’ of urban space has become common practice in Harare’s CBD, where state and municipal police officers collaborate to punish what they perceive as ‘spatial unruliness’. This aggressive repression is often legitimized as an intervention to ‘purify’ and decongest prime city spaces. The argument of the City of Harare is that street traders create an unfavourable environment for formal businesses and other city residents and thus deserve to be forcibly evicted to maintain order. More commonplace are everyday eradication strategies involving low-level harassment of vendors by “predatory” state and municipal enforcement officials, as narrated by some street traders and officials during our study.

I was once harassed by the law enforcement officers, and it was terrible. I was verbally and physically assaulted. They called me names and threatened to arrest me and told me that I should not be selling in the streets, and I should go back to the village where I came from. They took my wares and destroyed them. (Female Street trader, FGD)

The understanding which has been used by the local government is that these are illegal traders who are putting dirty in the streets, who do not obey the by-laws of their cities and they must be punished for that. So, you find that in Harare, they have always been harsh instruments, harsh declarations coming from the local authority or the metro level to deal with the street traders. As we speak now, there is an operation which was set up, which they are saying its an operation to restore order in the CBD. Under that operation, the target are street vendors trading on pavements etc., all of them should be removed and arrested. (Civil Society Leader 2)

The implementation of oppressive policies was acknowledged by urban governance actors. Municipal officers particularly planners and those from development control (e.g., enforcement agents) stated that they are obligated to act against street vendors to maintain order and clean up what they perceive as a nuisance and pathological urban practices.

In terms of urban practices, what we have had in Harare for some time now is the “cat and mouse” game between the authorities’ vendors; with the intention to bring order in the City Center. In most cases, the state collaborates with the City to implement operations aimed at restoring order in the CBD, because we cannot let everyone trade wherever they see it. We clean up urban spaces so that the city is not overtaken by street vending. Sometimes, the vendors turn the city upside down because they create several problems such as blocking traffic and pedestrian flows. (Principal Town Planner)

Our stance as a city is clear. The city operates guided by the by-laws and the laws of the land. So, you cannot have people selling on the streets. You cannot lock the movement of traffic. You cannot have a pushcart in the streets. You can only sell when you have a hawkers’ license. You can only sell when you have a permit. (City Planner)

From the planners’ perspective, street traders are violating planning regulations and thus, should be removed from the streets. Many street traders have highlighted significant losses of income when their goods are confiscated and destroyed during raids and operations.

Once your goods are confiscated, the whole business can potentially crumble, and you may struggle to bounce back. For me, my wares have been confiscated multiple times, sometimes without recovery. I have experienced this more than three times, getting goods confiscated and having to start from scratch. So, you can imagine the struggle and pain I experienced then. (Wainda)

Aggressive repression not only disrupts street traders’ daily operations but also creates a cycle of poverty and despair. Moreover, the use of violent crackdowns often results in a lack of trust and cooperation between street traders and local authorities. This further exacerbates the situation, as street traders are less likely to comply with regulations when they feel they are being treated unfairly. While the consequences of aggressive repression are collective, there are disproportionate implications of such a governing mode.

### Differential implications of aggressive repression

When examining the effects of aggressive policing, it becomes evident that the experiences of street traders are not uniform. Female street traders, for example, face additional challenges and vulnerabilities due to deep-rooted gender inequalities. One of the most visible manifestations of differential implications of the City’s aggressive repression is the double burden of precarious work and childcare responsibilities. In Harare’s public spaces it has become common to come across scenes of mothers with small babies (‘*street vending mothers*’) selling different types of goods. These women have few options and are forced to make difficult decisions to provide for their families. The use of makeshift cribs also puts the babies at risk, as the boxes or crates may not be stable or secure enough to ensure the safety of the child.

Women have realised that it is safe to put their babies in the boxes because if anything happens (e.g., raids), the baby will remain in the box. You just carry the box and run with the baby. If you have few things, you can just put them in the box together with the baby and run away. As a mother you must protect your baby and at the same time you have to protect your goods from confiscation and protect your livelihood. (Susan)

During enforcement and surveillance street vending mothers must make difficult choices between caring for their children, attending to customers and protect their goods from confiscation. To street vending mothers, these are not easy decisions to make because by virtue of being a woman, they are perceived to have the primary duty of taking care of their children. However, majority of the women selling in the streets of Harare have no choice but to deal with this reality since they are in a precarious financial position. Furthermore, the differential burden of hostile policing falls heavily on the elderly and street traders with disabilities, thereby magnifying pre-existing disparities.

The council officers are very violent and inconsiderate. They do not even consider that I am old women and a widow who is trying to make survive in a very difficult situation. I have a broken arm as you can see here, I was harassed by the council officers. They knocked on the ground with my hand first. I woke up later that day unsuspectingly, in Parirenyatwa Hospital. The council officers took all my wares. I went broke. I have 4 children to feed, and they need to go to school. They were so ruthless, and I wouldn’t be wrong if I say they wanted me dead. I don’t like the council not even the slightest bit. They broke my arm, and I became disabled. (Martha)

For disabled people, some of the enforcement officers are considerate and spare them. However, some of them do not care about that, and will tell you that no one is above the law. If you look at this guy, he is disabled but sometimes the council officers confiscate his goods and sometimes he has to pay fines to get them back. For women, most of them are single mothers who must survive and when their livelihoods are disrupted, they resort to negative coping mechanisms such as prostitution. (Tinashe)

The insights gathered from participants reveal that street traders with disabilities encounter unique obstacles in their daily lives. Disabilities imposes limitations on mobility, or physical endurance, making individuals more susceptible to aggressive policing. The lack of sensitivity, understanding, and accommodation from some law enforcement officers exacerbates the challenges faced by traders with disabilities, amplifying their vulnerability and hindering their ability to engage in economic activities freely. It is evident that the current policy and legislative approach to street trading in Harare fails to sufficiently consider differential vulnerabilities experienced by traders, as highlighted by a Senior Municipal Police Officer:

You see the by-law itself does not differentiate between disabled and abled person. The discretion lies with the enforcement officers… there is no provision for such in the by-laws. But, on humanitarian grounds, those things can guide the officers to rethink their actions against disabled vendors for instance. Even if goods belonging to a blind person have been confiscated, there is no law that tells us to return those goods without the person paying the administration fees.

From the above sentiments, it is evident that municipal policy instruments are designed with limited, or no consideration given to how their deployment is experienced by different groups of street traders. However, the enforcing officers do have some leverage and discretion based on humanitarian grounds. Unfortunately, female street traders have reported instances of violent harassment by municipal police officers, who show no consideration for factors such as age, disability, and other conditions. Despite the persistent implementation of punitive crackdowns on street traders in Harare, it has become increasingly evident that eliminating their presence is an unattainable goal. The combination of inadequate enforcement mechanisms and the worsening economic conditions in the city has hindered any meaningful progress in this regard. Aggressive repression implies the conflicts between enforcement authorities and street traders. Given these challenges, the City of Harare has experimented with an alternative mode of governing through creating designated trading spaces.

### Governing street trading through spatial containment: “*They should operate in designated vending sites*”

The City of Harare has experimented with an alternative governing mode, which involves specific designated sites where street traders are expected to operate. We characterize this approach as governing through ‘spatial containment’. This mode of governing street trading has two specific objectives. First, to ‘tame’ the problem of street trading by restricting their operations to designated sites, both within and outside the central city area. Spatial containment might be interpreted as a form of ‘spatial distancing’, where urban authorities transfer the ‘problem’ into enclosed marketplaces in the name of maintaining order and urban aesthetics. Second, by creating these designated spaces, the city seeks formalize street trading.

These street traders are not supposed to be where they are. We have created designated places for them to operate from there. The idea is to formalize street trading and ensure urban order. (City Planner).

Our position as a city is that vendors should operate within a Legal Framework in which they will be accommodated in urban spatial plans and governance frameworks. This is why we have been creating designated vending sites to accommodate those that would have been displaced from the central city areas during ‘clean up' operations. We are also exploring options to create spaces where people can be allowed to vend. (Principal Town Planner).

Although Harare’s Small and Medium Sized Enterprises (SMEs) Policy of 2023 suggests that the allocation of trading space shall be inclusive to cater for all groups (e.g. women, youth and people living with disabilities), the reality is different. The decisions regarding who gets to stay in the designated sites are primarily made by municipal authorities (through the SME committee) and are largely influenced by political considerations, rather than a transparent or equitable process as highlighted by one of the participants:

I do not even know how someone like me is supposed to access the designated places. I was not part of any meeting to share my views. So, what happens is that the city officials just decide what they think is appropriate and make announcements that vendors will be moved to a particular place. As for who is expected to benefit, I must be honest with you. Most of the stalls in these places belong to city officials and politicians. (Tinashe)

…the reason is because some key decision-making spaces are being occupied by people who have selfish ideologies, and they have that power to influence…. (Civil society leader 2)

In my view, designated sites are being allocated by the ruling party, giving preference to their members. There is a lot of politics in these processes. Therefore, the city of Harare must create room for all street traders and allocate trading spaces equally without bias…. (Civil society leader 4)

Tinashe’s sentiments suggests that the planning and decision-making processes do not adequately consider the needs and circumstances of all vendors. This is further emphasized by the sentiment, “*I was not part of any meeting to share my views*”*,* indicating a lack of participatory processes in decision-making. Vendors feel that decisions are made unilaterally by city officials without consulting those who are directly affected. The second quote from the civil society leader, “...*some key decision-making spaces are being occupied by people who have selfish ideologies, and they have that power to influence*”, reinforces the notion that the planning and implementation of spatial containment are influenced by political considerations. It points to a broader issue of governance where decision-making is dominated by individuals with vested interests, further marginalizing the vendors and limiting their ability to benefit from these initiatives. Governing through spatial containment has significant implications for street trading. The establishment of designated vending sites has the potential to undermine key attributes of street trading, including proximity to consumers, spatial mobility, and operational flexibility. Consequently, when street trading is governed through spatial containment measures, there is a risk that vendors may choose to abandon the designated sites.

The city council do not respect us at all. We cannot move to the bays they constructed outside the city. We want to be allocated space somewhere within the city center where there is market. We also want free spaces because some of us cannot afford to pay rents. (Adonia, male street trader in an FGD)

The city must not allocate space far away from our market base because that alone will not work. For example, who do you expect to come to buy fruits and vegetables at Coca Cola vending site? How about the city just charge me from where I am anchored because that is where my customers always find me? I do not support this idea of moving vendors to spaces that are outside the city center. (Emmah, Female street trader)

If you look at the Coca cola vending site, it is far away from the city center, and business is very low there. Customers are in the CBD, there are buildings which are not being utilized and if the authorities give us these buildings, and put water and toilets, traders are ready to operate there and work. A good example is the Copacabana area, it is very conducive, and customers can easily go there, and so there is business …. Any places that are out of town are not favorable to us as traders since there are no customers …. (Civil society leader 4)

The above sentiments demonstrates that the designated sites outside the CBD are not suitable for street traders’ businesses. Instead, they request the allocation of market spaces within the city center where there is spatial proximity to their market base. Willard, a 33-year-old itinerant street trader, who sells also expressed his frustration at the City’s plans to relocate vendors to designated places outside the central city area:

For me, this idea of trying to put me into walls and immobilize my business does not work. I run my business by moving from place to place looking for customers at busy places such as bus termini and pedestrian crossings. How am I supposed to operate if I am moved to a building somewhere outside the city center? I will lose money and will not be able to easily connect with my customers.

The above quote suggests that governing street trading through spatial containment is a form of ‘*forced conversion*’ (Kamete, 2013b)*,* which undermines street traders’ modus operandi. Street traders often rely on the ability to adapt their operations based on changing market dynamics, customer demands and municipal enforcement rhythms. Therefore, spatial containment may curtail some of these operational dynamics, thereby restricting the adaptability of street traders.

## Street traders’ associations in policy and urban governance

While policies and governance practices towards street trading in Harare are predominantly centered around state control, there has been a notable rise in the formation of street traders’ associations aiming to represent the collective interests of vendors in urban affairs. These associations, such as the Vendors Initiative for Socio-economic Transformation (VISET), National Vendors Union of Zimbabwe (NAVUZ), Zimbabwe Chamber of Informal Economy Association (ZCIEA), among others, play a significant role in advocating for the rights of street vendors. For instance, VISET has been instrumental in the establishment of the informal sector working group in the City; a platform that brings together different stakeholders to dialogue on issues affecting informal sector workers.

However, a major challenge faced by the street traders’ associations is the lack of legal recognition within the laws of Zimbabwe. This lack of recognition undermines their efforts to effectively defend the collective interests of their members. Without legal status, they face limitations in terms of engaging in formal dialogue with relevant authorities, accessing resources, and participating in decision-making processes. The relationship between street traders’ associations and the city is complex and multifaceted. Ongoing conflicts regarding representation and the authority to speak on behalf of street vendors often result in tense situations, and some association leaders have been subjected to violence. In a politically polarized environment, street traders’ associations are sometimes viewed as agents of regime change, leading to accusations that their intentions are to destabilize the government. These discourses further complicate the establishment of meaningful relations between the associations and urban authorities, hindering constructive dialogue and collaboration.

…these organizations are often perceived to be linked to politics; this makes their sustainability and operations vulnerable. For instance, if VISET is to organize a meeting with vendors, that meeting will be disrupted by the police because it will be perceived as political mobilization. So, this context makes it difficult to organize and have a collective voice in the affairs of the city. (Interview with Tinashe)

Street traders’ organizations in Harare have a long history of fragmentation, which weakens their collective influence in urban governance processes. While some individual organizations like VISET are witnessing positive strides in their engagement with urban authorities, their efforts have not resulted in significant changes in policy and governance practices towards street trading. Interviews with street traders brought out allegations that some leaders’ management of associations is based in personal political ambition or economic interest, while others collect money in the guise of subscription fees to allocate secure spaces in markets or on streets and to protect street traders from police harassment, with no traceable activities on the ground. It is such structural bottlenecks that continue to undermine the influence of street traders associations in governance processes. Furthermore, the politics of polarization affects the effectiveness of street traders’ associations in influencing policy and governance processes.

…Because there were a lot of associations that were politically aligned; they were those that were affiliated to the ruling party, ZANU PF and others that were aligned to the opposition. This created problems in terms of rationalizing their activities as they could often fight among themselves…. (Chief Development Control Officer, City of Harare)

The political affiliations of these associations further weaken the prospects of collective bargaining with policymakers. Instead of a unified body representing street traders’ interests, there are multiple groups with competing political loyalties. This division can lead to conflicts among the associations, as they may prioritize their political agendas over the collective interests of street traders.

## Discussion and conclusions

This paper examined how urban authorities in Harare respond to street trading and the implications of these interventions on street traders’ access to urban space. Our findings demonstrate that Harare’s pursuit of modernistic aspirations is defended by highly technocratic and repressive planning systems that are activated and mobilized to push anti-poor policies. This form of ‘*repressive managerialism*’ is intensifying in many Global South cities, where authorities are determined to eradicate street traders’ livelihoods (Kamete, 2013a; Lindell, 2019). As our findings suggest, the planning and governance architecture in Harare persistently displays its dark side against the urban poor (Watson, 2009), who are engaging in legitimate livelihood activities. In Harare and indeed most cities in the Global South, there is a considerable gap between designed cities—as established in legislation and planning systems—and the ‘actually’ existing city that is produced and reproduced by citizens in everyday life (Kamete, 2013b; Watson, 2009). The defence of a modern city project is often accompanied by interventions of a ‘sanitizing’ character with devastating consequences for the urban poor (Bandauko et al., 2024; Rogerson, 2016).

The ‘sanitization’ of urban spaces has spread rapidly in the Global South. In China, the ‘global city’ aspirations have resulted in abusive policing and violent encounters between street traders and law enforcement authorities (Hanser, 2016; Huang et al., 2014). In Mexico city, a historic center revitalization program resulted in large scale displacement and removal of street traders, in order to create a ‘positive’ image of the city (Crossa, 2009). African cities, such as Accra (Ghana), entrepreneurial urban governance centered on attracting private capital trigger dispossession of the urban poor, thereby undermining socio-spatial justice and inclusive urban development (Gillespie, 2017). As demonstrated in our study, aggressive repression prefers a governing norm that emphasizes legality, modernity, and image building at the expense of economic needs and welfare of the urban poor (Recio et al., 2017). Planning as an instrument of the state is mobilized and used as spatial technology of domination to impose ‘order’ onto urban space and erase any visible signs of informality (Lindell et al., 2019). Aggressive repression often results in a violation of the basic human rights of street traders, who are denied their right to physically occupy and appropriate urban spaces and participate in the urban economy (Bandauko and Mandisvika, 2015).

Although the experiences from Harare reveal a largely antagonistic approach to street traders, there are some progressive approaches that have been documented in other cities. For instance, the district police officers (the *thetsakit*) in Soi Rangnam, Bangkok do not actively try to eradicate the livelihoods of unregistered mobile vendors: a scenario referred to as “*managed informality*” (Batréau and Bonnet, 2016). The district administration in this context does not consider street vending as a nuisance, but rather individuals who are running their businesses to make ends meet. This approach recognizes the economic necessity of street vending and aims to balance regulation with the vendors’ need to sustain their livelihoods.

As Lindell et al. (2019) argue, different governing approaches may be differentially deployed upon different spaces and groups; thus, generating variegated experiences among diverse groups of street traders. Our findings also reveal that Harare’s authoritarian spatial governance is disproportionately experienced, demonstrating that street traders are “internally differentiated, resulting in different degrees of exclusion, power, resources, mechanisms of exclusion…” (Crossa, 2020: 170). For example, women, especially ‘*street vending mothers*’ struggle to balance between caring for their children, attending to customers and watching for and evading enforcement authorities (Bandauko et al., 2024). Although some enforcement officers have discretionary powers to spare some vulnerable street traders such as those with disabilities and the elderly, participants indicated that in most instances these groups also endure violent crackdowns, harassments and confiscation of their merchandise. In Harare, while most street vendors operate informally, there are instances where permits are issued, creating a semi-formal status. This is the case for those who operate in designated places. These vendors face different levels of enforcement compared to those without permits, indicating a spectrum rather than a dichotomy. However, the processes of acquiring a permit are cumbersome, leaving majority of the street traders to operate informally. In fact, all the participants in our study were not licensed and this makes them highly susceptible to violent enforcement irrespective of their socio-economic status.

This situation is somehow contrary to what has been observed in other cities. In Bangkok, some vendors receive differential treatment from the police officers based on their socio-economic status (Pulliat et al., 2024). For instance, older street traders and those with children are spared from municipal enforcement and strict surveillance (Pulliat et al., 2024). In Harare, this form of lighter enforcement is not extended to all underprivileged street traders. In our research, we heard very disturbing stories of street vending mothers who were violently harassed, arrested and their good confiscated despite their double burden of childcare and precarious informal work. Therefore, the “mutually constitutive gender ideologies of space and work produce unequal conditions” (Dunn, 2015: 25) for male and female street traders. Gender is a salient social condition that impacts on different aspects of street trading including experiences with municipal enforcement policies and practices (Adama, 2020; Bandauko et al., 2024; Fåhraeus, 2024). This highlights the gendered nature of access to and appropriation of urban space (Crossa, 2016, 2020; Fenster, 2005), wherein the consequences of aggressive spatial governance are experienced in distinct ways.

Aside aggressive repression, Harare has experimented with spatial containment as alternative governing mode. Spatial containment is aimed at immobilizing street traders in designated areas to address concerns such as disorder and the need for modernization of informal enterprises (Kazembe et al., 2019). This strategy is meant to “fix street vendors within walled spaces and render them visible targets for mechanisms of control, and normalisation…” (Lindell et al., 2019: 78). To a certain extent, spatial containment is considered by city officials as a progressive model due to its alignment with the government’s agenda of promoting the formalization of small to medium enterprises. Similar to Harare, the Bangkok Metropolitan Administration (BMA) was determined to move street traders to designated marketplaces located in the outskirts of the city (Boonjubun, 2017). In Harare, there are conflicting rationalities between the city’s plans to relocate vendors to designated marketplaces. To the officials (e.g. planners, policymakers and politicians), moving vendors to designated sites is an approach to reorganize urban space and create an environment that uphold the beauty and image of the city. Thus, spatial containment represents the logic of aesthetic governmentality (Ghertner, 2010), which denotes governmental practices that are based on an evaluation of the aesthetic appropriateness of those governed, particularly street traders, in accordance with a predefined set of aesthetic standards intended to transform Harare into a world-class city (Bandauko and Arku, 2024a). To the street traders, urban space is a form of ‘livelihood space’, which provides memories of years of struggles for survival in a harsh economic environment. While relocating street traders to designated sites may align with officials’ goals of creating a more organized and visually appealing city, it often comes at the expense of inclusivity and access to urban space for marginalized groups.

While moving street traders to off-street locations may be relatively straightforward, the challenge lies in attracting and redirecting their customer base to these new areas. The difficulties in successfully implementing this approach highlight the complexity of the spatial politics surrounding street trading. Simply moving street traders to designated enclaves does not guarantee the desired outcomes of order and modernization. Studies conducted in other Global South cities also confirm that moving street traders to designated sites might not be an effective governing mode if issues such as spatial proximity to customers are ignored. In Guangzhou, China, a study conducted by Huang et al. (2019) discovered that when traders were relocated to designated sites, they experienced a 20% decline in customer volume compared to when they were vending in open spaces. Thus, spatial containment of street traders breaks down the organic connection between street traders and their potential customers, thereby compromising the ability of traders to engage with their target market. Kamete (2018) describes measures such as spatial containment as ‘*pernicious assimilation*’ of economic actors, which might have unintended outcomes. Through pernicious assimilation (Kamete, 2018), street traders are expected to conform to formal regulations and norms to gain legitimacy but experience detrimental outcomes such as the potential to lose their customer base as stated earlier on. Consequently, most of the designated vending sites outside the Harare CBD have been abandoned as street traders retreated to central city areas, as reported in other African cities like Kumasi, Ghana (Akuoko et al., 2022). Another important dimension is the politics of accessing designated vending sites under the model of spatial containment. A study conducted by Oosterom and Gukurume (2022) reveals that political patronage networks are influential in determining who has access to vending spaces in Harare. Thus, allegiance to a political party becomes the first criterion for one to access some of these contested trading spaces (Oosterom and Gukurume, 2022). In most cases, access to urban space is manipulated by rent-seeking behaviour, often excluding those who rightfully deserve to benefit.

While other scholars have observed the significant influence of street traders’ associations in governance processes in other cities like Accra (Gillespie, 2017), Maputo (Lindell, 2008) and Cusco (Steel, 2012), in Harare street traders’ collective engagement with the state is limited. In India, street traders have registered some notable progress in challenging exclusionary policies through the support of NGOs such as the Self-Employed Women’s Association (SEWA) (Roever and Skinner, 2016). In Africa, experiences from Dakar, Senegal reveal that strategic collective mobilization and organizing enhanced demonstrations which resulted in street traders gaining political rights and spatial legitimacy (Brown, 2015). In countries like Malawi, Zambia and Mozambique, there is an enduring process where street traders are coming together and create a system that advocates for their rights across time and space (Lindell, 2008). The work of street traders’ associations in Harare is constrained by a heavily regulated civic space, which limits their influence in urban decision-making processes. Civic space in Zimbabwe is rapidly shrinking as the government continue to thwart dissenting voices through repressive legislation. The Government of Zimbabwe recently passed the Private Voluntary Organizations (PVO) amendment bill which restricts the activities of civil society groups in politics and governance. The limited avenues for participation and the restricted freedom of expression and association in Harare hinder the ability of street traders’ associations to advocate for policy changes that address the needs and concerns of street traders.

This paper contributes to and advances scholarly discourse on the complex dynamics between urban governance, informal livelihoods, and policy frameworks in the context of street trading. We present a nuanced and complex narrative that sheds light on the disproportionate impacts of aggressive spatial governance, demonstrating that street traders as a heterogenous social group experience policy differently. Empirically, our research provides valuable insights into the specific policy and governance responses to street trading in Harare. By conducting in-depth interviews and focus group discussions, we gather firsthand data on the experiences and perspectives of street traders, as well as the strategies employed by policymakers and planning institutions. The findings have implications for urban policy and practice, providing valuable insights into the challenges and opportunities related to street trading in urban environments. These implications can inform the development of more effective and responsive policies that address the complex dynamics associated with street trading and promote inclusive urban development. In the implementation of spatial formalization policies, urban governments should create platforms for dialogue so that there is a win-win situation in how urban space is produced and appropriated. Urban authorities should engage in participatory planning processes that involve street traders and their representative organizations. It is essential for urban planners and policymakers in African cities to incorporate the issue of urban informality into broader discussions on social justice (Kamete, 2013a). By doing so, they can address the inherent inequalities and challenges faced by informal workers. All of this will require political will and sustained commitment to make cities inclusive and friendly to the needs and aspirations of the marginalized groups like street traders. We agree with Martínez et al., (2017: 25) who argue that “any policy intervention should take into account the important variation within and amongst street vendors”. As our findings show, street traders are not a homogeneous group; they vary widely in terms of the goods they sell, the scale of their operations, gender, age and their socio-economic backgrounds. Policies that fail to consider these differences may not be effective in addressing the specific needs and challenges faced by different groups of vendors.

The findings presented are also relevant for street traders’ associations. For street traders’ associations to be effective in influencing policy and governance practices in Harare, they may need to reconsider their current state of organization, since their level of fragmentation hinders their bargaining power. Given the structural forces street traders are up against, it is critical to build counter power and strengthen collective bargaining. Without a consolidated front, these associations struggle to challenge exclusionary practices, ultimately limiting their influence on urban governance and decision-making processes (Bénit-Gbaffou, 2016). Therefore, we propose the establishment of a consolidated national association of street traders that transect patronage dynamics. Experiences from India demonstrate that a collective national voice of street traders was instrumental in the promulgation of the Street Vendors (Protection of Livelihood and Regulation of Street Vending) Act, 2014 (Roever and Skinner, 2016), which protects livelihoods of the poor and compels municipalities to adopt participatory decision-making processes. By establishing a cohesive national association, street traders in Harare could similarly advocate for policies that protect their livelihoods and promote inclusive urban governance.
